# Seroprevalence of *Toxoplasma gondii* infection and risk factors in sheep from Afyonkarahisar province, Türkiye

**DOI:** 10.3389/fvets.2026.1912990

**Published:** 2026-08-14

**Authors:** Ahmet Cihat Tunç, Mahmut Sinan Erez, Esma Kozan, Fatih Mehmet Birdane, Luis Pablo Hervé-Claude

**Affiliations:** 1 Department of Internal Medicine, Faculty of Veterinary Medicine, Afyon Kocatepe University, Afyonkarahisar, Türkiye; 2 Department of Parasitology, Faculty of Veterinary Medicine, Afyon Kocatepe University, Afyonkarahisar, Türkiye; 3 Department of Clinical Sciences, Lewytt College of Veterinary Medicine, Long Island University – Post Campus, Brookville, NY, United States

**Keywords:** abortion, barn cats, biosecurity, communal grazing, farm cats, one health, sheep, *Toxoplasma gondii*

## Abstract

**Background:**

*Toxoplasma gondii* is a major zoonotic pathogen causing reproductive losses in sheep and posing significant public health risks, particularly through meat consumption and congenital infection. Despite Afyonkarahisar Province being one of the most important sheep-producing regions in Türkiye, no farm-level seroepidemiological study with structured risk factor analysis had previously been conducted there.

**Methods:**

A cross-sectional study was conducted between September and December 2025 on 550 sheep blood samples collected from 55 commercial farms across Afyonkarahisar Province. Anti-*T. gondii* antibodies were detected using a commercial indirect ELISA (ID Screen^®^ Toxoplasmosis Indirect Multi-Species, IDvet), with samples classified as positive (sample-to-positive percentage [S/P%] ≥ 50%), doubtful (40%−50%), or negative (≤40%). A structured questionnaire was administered to collect 19 farm- and animal-level variables. Associations with seropositivity were evaluated by univariable analysis and a mixed-effects logistic regression model with farm as a random effect, with Firth's penalized regression applied to obtain stable estimates where quasi-complete separation occurred.

**Results and Discussion:**

The overall animal-level seroprevalence was 42.2% (232/550; 95% CI: 38.0%−46.4%), and 44 of 55 farms (80.0%; 95% CI: 67.0%−89.6%) were classified as seropositive. In multivariable analysis using Firth's penalized logistic regression, five variables were independently associated with higher seropositivity: flock-level history of abortion (aOR = 3.77; 95% CI: 2.41–6.00), age over 12 months (aOR = 3.54; 95% CI: 2.15–5.97), communal pasture use (aOR = 74.12; 95% CI: 10.22–9431.54, wide owing to quasi-complete separation), leaving aborted materials on site (aOR = 2.30; 95% CI: 1.49–3.61), and irregular barn cleaning (aOR = 2.32; 95% CI: 1.46–3.72). Cat presence was universal across all 55 farms, precluding statistical evaluation; however, stray cats had unrestricted access to feed and water on every farm, likely representing the primary driver of environmental oocyst contamination. Breed, sex, water source, and antiparasitic drug type were not significantly associated with seropositivity. *T. gondii* infection is highly endemic in sheep in Afyonkarahisar, with seroprevalence exceeding the national average. Stray cat access control, safe disposal of aborted materials, and routine differential diagnosis for abortifacient pathogens are recommended as priority interventions for reducing infection pressure and mitigating both animal health and public health risks within a One Health framework.

## 1 Introduction

*Toxoplasma gondii* is an obligate intracellular apicomplexan protozoan parasite with a worldwide distribution, capable of infecting all warm-blooded animals, including humans (1, 2). Among livestock species, sheep represent one of the most important intermediate hosts, as infection is strongly associated with abortion, fetal resorption, stillbirth, and neonatal mortality (3, 4). A recent global meta-analysis estimated a pooled seroprevalence of approximately 34% in sheep, increasing to 41% in European flocks (5). These infections result in substantial economic losses through both direct reproductive failure and indirect effects such as reduced fertility and milk production (6, 7). Moreover, modeling studies have demonstrated that even moderate increases in flock seroprevalence can significantly reduce lamb output, highlighting the cumulative production impact of endemic infection (8).

The life cycle of *T. gondii* is heteroxenous, with felids serving as the definitive hosts responsible for shedding environmentally resistant oocysts (1). Sheep typically acquire infection through ingestion of sporulated oocysts contaminating pasture, feed, or water sources (4). These oocysts can remain infective for extended periods under favorable environmental conditions, particularly in moist and temperate climates (9). Consequently, farm management practices that influence environmental contamination, such as grazing systems, water sources, feed storage, and cat population control, play a central role in shaping infection dynamics at the flock level (6, 10). Based on this transmission pathway, it is expected that variation in these management factors contributes substantially to the heterogeneity of infection observed between farms.

Beyond its veterinary importance, *T. gondii* is a major zoonotic pathogen. Human infection occurs primarily through consumption of undercooked meat containing tissue cysts or ingestion of oocysts from contaminated environmental sources (1). It is estimated that 30%−50% of the global human population carries a latent *Toxoplasma* infection (11). Of particular concern is primary infection during pregnancy, which may result in congenital toxoplasmosis and severe fetal outcomes, including neurological and ocular damage (12). In Türkiye, seroprevalence among pregnant women averages approximately 36.76% at the national level, with regional variability indicating ongoing exposure risk (13, 14). Given the role of small ruminants as a source of human infection, monitoring *T. gondii* in sheep populations is therefore essential within a One Health framework.

In Türkiye, multiple regional studies have reported *T. gondii* seroprevalence in sheep, with values ranging from below 5% in colder, high-altitude regions to considerably higher levels in warmer areas, reflecting the influence of environmental conditions and management practices on transmission (10, 15, 16). A recent nationwide study estimated an overall seroprevalence of 30.51% and highlighted substantial heterogeneity in infection dynamics across the country (17). However, most available studies in Türkiye have primarily focused on descriptive seroprevalence, with limited integration of structured farm-level epidemiological data to identify underlying risk factors.

Afyonkarahisar province, located in Central-Western Anatolia, represents one of the major sheep-producing regions of Türkiye, with an estimated population of approximately 940,000 sheep (18). The region is characterized by extensive grazing systems, widespread presence of stray and domestic cats, and traditional livestock management practices, all of which may influence environmental exposure to *T. gondii*. Despite its epidemiological relevance, data from this region remain limited. To our knowledge, no peer-reviewed study combining seroprevalence estimation with structured risk factor analysis has been conducted in Afyonkarahisar.

The present cross-sectional study therefore aimed to: (i) determine the seroprevalence of *T. gondii* infection in sheep from 55 farms in Afyonkarahisar province, Türkiye, using a commercial indirect ELISA and (ii) identify farm- and animal-level factors associated with seropositivity through a structured epidemiological questionnaire. This specific serological method was chosen over other diagnostic procedures due to its well-documented high sensitivity and specificity, excellent standardization, and robust applicability for large-scale epidemiological screening, which aligns perfectly with the objectives of obtaining reliable flock-level data. By integrating serological and management data, this study seeks to provide a more comprehensive understanding of the epidemiology of *T. gondii* in a major sheep-producing region and to support the development of targeted control strategies.

## 2 Materials and methods

### 2.1 Study design and study area

**Figure 1:**
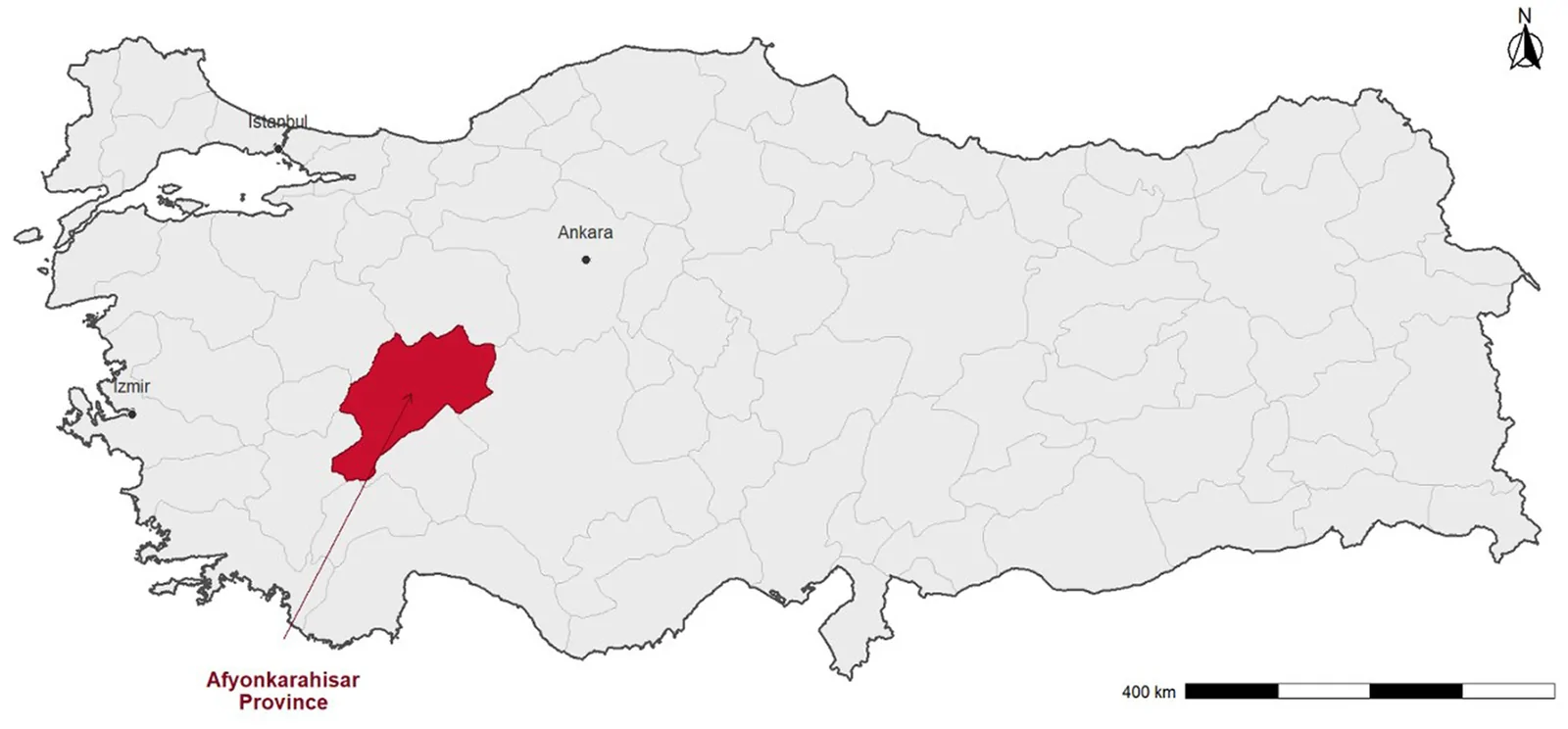
Geographic location of Afyonkarahisar province within Türkiye.

A cross-sectional seroepidemiological survey was conducted to estimate the seroprevalence of *Toxoplasma gondii* infection in sheep and to identify associated farm- and animal-level risk factors in Afyonkarahisar Province, located in the Inner Aegean Region of Türkiye (38°44′N, 30°32′E; altitude approximately 1,020 m above sea level) (Figure 1). The province has a semi-arid continental climate with cold winters and warm summers. Afyonkarahisar is one of the most important sheep-producing provinces in Türkiye, supporting a large sheep population under extensive and semi-extensive production systems characterized by communal pasture use and a tradition of local fresh meat and dairy consumption (19). Field sampling was carried out between September and December 2025.

### 2.2 Farm selection and sampling strategy

The study population comprised sheep maintained under commercial production conditions across Afyonkarahisar Province, Türkiye. Farms were eligible for inclusion if they: (i) maintained a minimum flock size of 30 sheep, (ii) had been in continuous operation for at least 1 year, and (iii) provided written informed consent from the farm owner prior to participation.

Farms were enrolled using a non-randomized convenience sampling approach. Farm owners were contacted primarily through the extensive professional network of the research team, which includes veterinarians working in government and private sectors as well as practicing field veterinarians within the region. Additional farms were recruited using a snowball sampling strategy, whereby participating veterinarians and enrolled farmers referred additional eligible flocks.

Although this sampling strategy has inherent limitations, including the potential for selection bias, the use of a non-randomized approach was considered appropriate due to the absence of a comprehensive province-wide sampling frame for sheep farms and the logistical constraints associated with extensive pasture-based production systems in the region. Efforts were made to include farms from different districts of Afyonkarahisar Province to maximize geographic representation and variability in management practices.

A two-stage cluster sampling design was applied. In the first stage, 55 farms were selected as primary sampling units. In the second stage, 10 sheep were sampled from each farm, yielding a total of 550 animals. Within each flock, animals were selected non-randomly to represent available age and sex categories as evenly as possible. Individual animal identifiers were recorded at the time of sampling to avoid duplicate inclusion of animals, when available.

### 2.3 Sample size calculation

The minimum required sample size for seroprevalence estimation was calculated using the standard formula for prevalence studies proposed by Thrusfield (20):


$$\begin{array}{l} \text{n}\;=\;\left(\text{Z}\;\times \;\text{P}\left(1\;-\;\text{P}\right)\right)\;/\;\text{d} \end{array}$$

Where *Z* = 1.96 for a 95% confidence level, *P* = 0.40 (expected prevalence based on prior national estimates) (10, 17), and *d* = 0.05 (desired absolute precision). This yielded a minimum of 369 animals. Because sheep were sampled within farms using a clustered design, the sample size was adjusted by a design effect of 1.5 to account for within-farm correlation, giving an adjusted minimum of 554 animals (20). Accordingly, 550 sheep distributed across 55 farms (10 per farm) were targeted and sampled, a figure consistent with the adjusted requirement.

### 2.4 Risk factor data collection

During each farm visit, flock- and animal-level epidemiological data were recorded using a standardized structured questionnaire administered to the farm owner or manager (Supplementary File S1). Farm-level variables included: total flock size, production purpose (meat, milk, or mixed), predominant breed (Pirlak, Merino, Akkaraman, or crossbreed), altitude category of the farm location, grazing system (full-day grazing of approximately 12 h or extended grazing exceeding 12 h per day), grazing duration in months per year, type of grazing area (communal or private), main roughage type (hay or straw), feed storage conditions (open or covered), water source (borehole/well water or surface/spring water), water delivery system, presence and number of cats on farm premises, cat access to feed storage and drinking troughs, presence of dogs on farm, barn cleaning frequency, method of disposal of aborted materials and placentae, use of rodent control measures, history of abortions in the flock, estimated flock abortion rate (%), number of abortions recorded in the preceding 12 months, use of antiparasitic drugs and treatment frequency, and the type of antiparasitic agent used (benzimidazoles or macrocyclic lactones). Animal-level variables included age category (under or over 12 months), sex, and physiological status. The 12-month age cut-off was specifically chosen as an epidemiological milestone to differentiate young replacement stock, which have had limited temporal exposure to the environment, from adult breeding ewes that have completed at least one full extensive grazing and reproductive cycle. The season of sampling was uniform across all farms (autumn) and was recorded as a fixed variable.

### 2.5 Blood collection and serum preparation

Blood samples of approximately 10 mL were collected aseptically from the jugular vein of each animal into vacuum clot-activator tubes. Samples were transported to the laboratory on the same day under refrigerated conditions (2–8 °C). Sera were separated by centrifugation at approximately 2,500 × g for 10 min, aliquoted into labeled 2-ml microtubes, and stored at −20 °C until serological analysis. Samples with visible haemolysis or lipaemia were excluded from analysis.

### 2.6 Serological testing

All serum samples were tested for the presence of anti-*T. gondii* antibodies using a commercial indirect ELISA kit (ID Screen^®^ Toxoplasmosis Indirect Multi-Species, IDvet, Montpellier, France). The assay was performed strictly according to the manufacturer's instructions. Optical density (OD) values were measured at 450 nm using a Thermo Scientific Multiskan FC Microplate Photometer (Thermo Fisher Scientific, Waltham, MA, USA). Validated positive and negative control sera supplied with the kit were included on each microplate, and plate validity was confirmed against the manufacturer's acceptance criteria before results were recorded.

Results were expressed as a sample-to-positive percentage (S/P%) calculated as:


$$\begin{array}{l} S/P\%\;\;=\;[(OD\;sample\;-\;OD\;negative\;control)\;/\\ \;\left(OD\;positive\;control\;-\;OD\;negative\;control)\right]\;\times \;100 \end{array}$$

Samples were classified as seronegative when S/P%≤40%, doubtful when S/P% was between 40 and 50%, and seropositive when S/P% ≥50%, in accordance with the manufacturer's guidelines and published applications of this kit in ovine seroepidemiological studies (9, 21, 22). Non-conclusive results were re-tested in duplicate; samples remaining doubtful after re-testing were classified as seronegative for the purposes of prevalence estimation, consistent with the conservative approach applied in comparable studies (22).

### 2.7 Statistical analysis

Animal-level seroprevalence was calculated as the proportion of ELISA-seropositive sheep among all tested animals, with exact binomial 95% confidence intervals (CI). Farm-level seroprevalence was estimated by classifying a farm as positive if at least one of the 10 sampled sheep tested seropositive. Associations between individual seropositivity and recorded risk factor variables were first explored by univariable analysis using Pearson chi-square tests, or Fisher's exact test where expected cell frequencies were below five. Crude odds ratios (OR) with 95% CI were calculated for each variable. Variables with *P* < 0.20 in univariable analysis were considered as candidate variables for inclusion in the multivariable model (23, 24). A mixed-effects logistic regression model was then constructed with seropositivity as the binary outcome and farm included as a random effect to account for the clustering of animals within farms. Collinearity among candidate variables was assessed prior to model construction using the variance inflation factor (VIF); variables with VIF > 5 were excluded. Model fit was evaluated using the Hosmer–Lemeshow goodness-of-fit test. Because the grazing-area variable exhibited quasi-complete separation, which precluded stable estimation under the random-effects model, the multivariable model was re-fitted using Firth's penalized maximum-likelihood logistic regression (25), implemented in the logistf package; the penalized estimates are reported as the final multivariable results. Statistical significance was defined as *P* < 0.05 throughout. All analyses were conducted in R (version 4.4.1; R Core Team) (26) using the ‘lme4′ package for mixed-effects modeling and the ‘logistf' package for penalized regression.

## 3 Results

### 3.1 Animal-level seroprevalence

Of 550 sheep serum samples tested by indirect ELISA, 232 were classified as seropositive (S/P% ≥50%), yielding an overall animal-level seroprevalence of 42.2% (95% CI: 38.0%−46.4%). A further 24 samples (4.4%) produced results in the doubtful range (S/P% 40%−50%); these were re-tested in duplicate and, as results remained equivocal, they were classified as seronegative. The remaining 294 samples (53.5%) were seronegative (S/P%≤40%). The full animal-level seroprevalence summary is presented in Table 1.

**Table 1:** Summary of *Toxoplasma gondii* seroprevalence in sheep from Afyonkarahisar Province, Türkiye (*n* = 550 animals, 55 farms) in 2025.

Parameter	*n*	Percentage (%)	95% CI
Animal-level results			
Total tested	550	100.0	—
Seropositive (S/P% ≥50%)	232	42.2	38.0–46.4
Seronegative (S/P%≤40%)	294	53.5	—
Doubtful (S/P% 40%−50%)^a^	24	4.4	—
Farm-level results			
Total farms sampled	55	100.0	—
Seropositive farms (≥ 1 positive animal)	44	80.0	67.0–89.6
Seronegative farms (all animals negative)	11	20.0	10.4–33.0
Mean within-farm seroprevalence (%)	—	42.2	—
Range of within-farm seroprevalence (%)	—	0.0–70.0	—
Median seropositive animals per farm	5	—	—

^a^Doubtful samples were re-tested in duplicate and classified as seronegative for prevalence calculations. 95% CI by Clopper–Pearson exact binomial method. CI, confidence interval.

### 3.2 Farm-level seroprevalence

At the farm level, 44 of 55 farms (80.0%; 95% CI: 67.0%−89.6%) were classified as seropositive, defined as having at least one seropositive animal among the 10 sampled per farm. The remaining 11 farms (20.0%; 95% CI: 10.4%−33.0%) returned no seropositive animals (Farms 7, 9, 12, 23, 25, 27, 37, 40, 45, 49, and 53). Among the 44 seropositive farms, the number of seropositive animals per farm ranged from 1 to 7 (mean 4.2 ± 1.9; median 5), corresponding to within-farm seroprevalences of 10.0%−70.0%.

No farm included in the study had conducted laboratory investigation of abortion events for other infectious causes, including *Neospora caninum, Brucella* spp., or *Chlamydia abortus*. Abortion history was recorded as reported by farmers or farm workers and was not laboratory-confirmed.

### 3.3 Univariable risk factor analysis

Associations between seropositivity and each of the 19 recorded variables were evaluated using Pearson's chi-square or Fisher's exact test. Five variables were statistically significant at *P* < 0.05: age category, type of grazing area, history of abortion, disposal of aborted materials, and cleaning frequency. Two additional variables, presence of dogs on farm and main roughage type, were also significant at *P* < 0.05 but were not retained as independent factors following multivariable analysis. All results are presented in Table 2. Variables with *P* < 0.20 were included as candidates for multivariable analysis.

**Table 2:** Univariable analysis of potential risk factors associated with *Toxoplasma gondii* seropositivity in sheep from Afyonkarahisar Province, Türkiye (*n* = 550) in 2025.

Variable	Category	*n*	Seropositive *n* (%)	OR (95% CI)	*P*-value
Age category^†^	Under 12 months (ref.)	110	26 (23.6%)	1.00 (ref.)	< 0.001
	Over 12 months	440	206 (46.8%)	2.86 (1.75–4.65)	
Type of grazing area^†^	Private (ref.)	40	0 (0.0%)	1.00 (ref.)	< 0.001
	Communal	510	232 (45.5%)	—^b^	
History of abortion^†^	No (ref.)	170	37 (21.8%)	1.00 (ref.)	< 0.001
	Yes	380	195 (51.3%)	3.79 (2.50–5.74)	
Disposal of aborted materials^†^	Buried (ref.)	410	155 (37.8%)	1.00 (ref.)	< 0.001
	Left on site	140	77 (55.0%)	2.01 (1.36–2.96)	
Cleaning frequency^†^	Weekly (ref.)	130	37 (28.5%)	1.00 (ref.)	< 0.001
	Irregular	420	195 (46.4%)	2.17 (1.40–3.36)	
Dog presence^†‡^	Yes (ref.)	470	189 (40.2%)	1.00 (ref.)	0.023
	No	80	43 (53.8%)	1.72 (1.07–2.76)	
Main roughage type^†‡^	Hay (ref.)	410	160 (39.0%)	1.00 (ref.)	0.010
	Straw	140	72 (51.4%)	1.67 (1.13–2.46)	
Sex	Female (ref.)	478	196 (41.0%)	1.00 (ref.)	0.150
	Male	72	36 (50.0%)	1.45 (0.88–2.40)	
Breed	Pirlak (ref.)	180	81 (45.0%)	1.00 (ref.)	0.117
	Merino	150	54 (36.0%)	0.69 (0.44–1.08)	
	Akkaraman	100	50 (50.0%)	1.22 (0.74–2.00)	
	Crossbreed	120	47 (39.2%)	0.80 (0.50–1.27)	
Physiological status	Pregnant (ref.)	336	133 (39.6%)	1.00 (ref.)	0.243
	Lactating	52	22 (42.3%)	1.11 (0.61–2.02)	
	Dry	162	77 (47.5%)	1.40 (0.97–2.03)	
Grazing system	Full-day ~12 h (ref.)	280	127 (45.4%)	1.00 (ref.)	0.125
	Extended > 12 h	270	105 (38.9%)	0.77 (0.55–1.08)	
Water source	Well/borehole (ref.)	320	126 (39.4%)	1.00 (ref.)	0.116
	Surface/spring	230	106 (46.1%)	1.33 (0.93–1.89)	
Feed storage	Open (ref.)	490	212 (43.3%)	1.00 (ref.)	0.142
	Covered	60	20 (33.3%)	0.65 (0.37–1.15)	
Cat presence type	Stray only (ref.)	440	187 (42.5%)	1.00 (ref.)	0.763
	Both (owned + stray)	110	45 (40.9%)	0.94 (0.61–1.44)	
Cats in housing	No (ref.)	470	206 (43.8%)	1.00 (ref.)	0.058
	Yes	80	26 (32.5%)	0.62 (0.37–1.02)	
Rodent control	Yes (ref.)	210	85 (40.5%)	1.00 (ref.)	0.524
	No	340	147 (43.2%)	1.12 (0.79–1.59)	
Antiparasitic drug type	Benzimidazoles (ref.)	380	154 (40.5%)	1.00 (ref.)	0.240
	Macrocyclic Lactones	170	78 (45.9%)	1.25 (0.86–1.81)	
Production purpose	Meat (ref.)	500	207 (41.4%)	1.00 (ref.)	0.240
	Mixed	50	25 (50.0%)	1.41 (0.79–2.53)	
Flock size^†^	Small (< 150 animals) (ref.)	80	26 (32.5%)	1.00 (ref.)	0.042
	Medium (150–249 animals)	300	123 (41.0%)	1.37 (0.78–2.41)	
	Large (≥250 animals)	170	83 (48.8%)	1.98 (1.14–3.46)	
Abortifacient testing	No (all farms)	550	—	—	—

OR, odds ratio; CI, confidence interval; ref., reference category. (the baseline group against which the odds of seropositivity in other categories are statistically compared). ^†^Statistically significant at *P* < 0.05. ^‡^Significant in univariable analysis but not retained in the multivariable model; association attributed to confounding with communal grazing and abortion history. ^b^OR not calculable due to a zero cell in the private-grazing group; Fisher's exact test applied. History of abortion is interpreted as a marker of prior T. gondii circulation rather than a causal risk factor, since abortion is a recognized consequence of infection. P-values were derived from Pearson's chi-square test or Fisher's exact test where expected cell frequencies were below five.

Of the 19 variables examined, seven were significantly associated with seropositivity in univariable analysis (*P* < 0.05): age category, type of grazing area, history of abortion, disposal of aborted materials, cleaning frequency, presence of dogs, and main roughage type; flock size was additionally significant (*P* = 0.042). The remaining variables including sex, breed, physiological status, water source, and the cat-related variables were not significantly associated with seropositivity. Full counts, odds ratios, and confidence intervals for all variables are presented in Table 2. The independent contribution of each candidate variable was assessed in the multivariable model described below.

### 3.4 Multivariable analysis

Variables with *P* < 0.20 in univariable analysis were entered into a multivariable logistic regression model with farm initially specified as a random effect to account for clustering of animals within farms. No evidence of problematic multicollinearity was detected (all VIF < 3.0).

Five variables retained statistically significant independent associations with *T. gondii* seropositivity in the multivariable model. Because all 40 animals from exclusively private-grazing farms were seronegative, the grazing-area term exhibited quasi-complete separation, which prevented stable estimation under the random-effects model. The model was therefore re-fitted using Firth's penalized maximum-likelihood logistic regression (25), which yields finite, stable coefficients under separation; the penalized estimates are reported here and in Table 3 as the final multivariable results. In this penalized model, history of abortion (aOR = 3.77, 95% CI: 2.41–6.00), age over 12 months (aOR = 3.54, 95% CI: 2.15–5.97), communal grazing (aOR = 74.12, 95% CI: 10.22–9431.54), disposal of aborted materials on site (aOR = 2.30, 95% CI: 1.49–3.61), and irregular cleaning frequency (aOR = 2.32, 95% CI: 1.46–3.72) were all independently associated with seropositivity (all *P* < 0.001). The wide confidence interval for communal grazing reflects the small number of private-grazing animals and the absence of seropositive cases among them; the lower bound of the interval was OR ≈ 10, and the univariable Fisher's exact test was also significant (*P* < 0.001).

**Table 3:** Multivariable Firth's penalized logistic regression for *Toxoplasma gondii* seropositivity in sheep from Afyonkarahisar Province, Türkiye (farm as random effect; *n* = 550) in 2025.

Variable	Category	aOR	95% CI	P value
History of abortion^†^	No (ref.)	1.00	—	< 0.001
	Yes	3.77	2.41–6.00	
Age category^†^	Under 12 months (ref.)	1.00	—	< 0.001
	Over 12 months	3.54	2.15–5.97	
Type of grazing area^†‡^	Private (ref.)	1.00	—	< 0.001
	Communal	74.12	10.22–9431.54	
Cleaning frequency^†^	Weekly (ref.)	1.00	—	< 0.001
	Irregular	2.32	1.46–3.72	
Disposal of aborted materials^†^	Buried (ref.)	1.00	—	< 0.001
	Left on site	2.30	1.49–3.61	
Dog presence	Yes (ref.)	1.00	—	0.234
	No	1.41	0.81–2.52	
Main roughage type	Hay (ref.)	1.00	—	0.943
	Straw	0.98	0.61–1.58	
Cats in housing	No (ref.)	1.00	—	0.714
	Yes	0.88	0.44–1.81	
Grazing duration	Full-day ~12 h (ref.)	1.00	—	0.234
	Extended > 12 h	0.77	0.50–1.20	
Water source	Well/Borehole (ref.)	1.00	—	0.323
	Surface/spring	1.24	0.82–1.93	
Feed storage	Open (ref.)	1.00	—	0.903
	Covered	1.06	0.41–2.72	

aOR, adjusted odds ratio; CI, confidence interval; ref., reference category. ^†^Statistically significant at *P* < 0.05. ^‡^The grazing-area term exhibited quasi-complete separation, as all animals from exclusively private-grazing farms were seronegative; the wide confidence interval reflects the small private-grazing subgroup, and the lower bound nonetheless indicates a robustly strong association. Estimates were obtained by Firth's penalized maximum-likelihood logistic regression to provide finite, stable coefficients under separation (25). History of abortion is interpreted as a marker of prior T. gondii circulation rather than a causal risk factor. The Hosmer–Lemeshow goodness-of-fit test indicated adequate model fit (*P* > 0.05); all variance inflation factors were < 3.0.

Dog presence, roughage type, cat presence in housing, grazing duration, water source, and feed storage conditions were not statistically significant in the adjusted model (all *P* > 0.05).

Model fit was adequate according to the Hosmer–Lemeshow goodness-of-fit test (*P* > 0.05). Complete multivariable results are presented in Table 3.

## 4 Discussion

*Toxoplasma gondii* is a globally distributed zoonotic pathogen of major veterinary and public health significance, with sheep among the most important intermediate hosts due to their susceptibility to gestational reproductive losses and their role as a source of human infection through meat consumption (1, 4, 6). Türkiye maintains one of the largest national sheep populations in the world, with over 42 million animals recorded in 2023 (18), and the sheep sector contributes approximately 20% of total national red meat production (17). Within this national context, Afyonkarahisar Province holds particular significance: located in the Inner Western Anatolia region at the intersection of the Aegean and Central Anatolian zones, it is recognized as one of the provinces with the highest sheep population density in the country (27, 28). Despite the province's livestock importance, farm-level investigations combining seroprevalence estimation with structured multivariable risk factor analysis for *T. gondii* had not previously been conducted in this area. To our knowledge, this is the first to do so, providing a provincial epidemiological baseline and identifying some key management-level determinants of *T. gondii* exposure in the sheep farming systems of this region.

### 4.1 Overall seroprevalence

The animal-level seroprevalence of 42.2% (95% CI: 38.0%−46.4%) and the farm-level prevalence of 80.0% (44/55 farms) confirm a high endemicity of *T. gondii* infection in Afyonkarahisar sheep. The provincial estimate exceeds the national mean of 30.51% reported in the first nationwide Turkish survey (17) and is broadly consistent with data from the Aegean and Central-Western Anatolian border regions (29). Across Türkiye, seroprevalence varies substantially, from 4.6% in the cold, high-altitude province of Erzurum (15) to considerably higher values in warmer and more humid areas, giving a national range of 9.5%−98.9% (10). At the international level, the 42.2% estimate falls within the range of recent IDvet ELISA-based studies: 39% in Australia (9), 53.8% in Italy (30), 38.1% in Iraq (21), and is also parallel to recent seroepidemiological findings reported in the broader Middle Eastern region (31), and 3.3% in the Faroe Islands, where low temperatures severely restrict oocyst survival (22). The use of the same commercial kits and identical S/P% thresholds across these studies enhances the validity of such comparisons. The 11 seronegative farms collectively shared lower-risk management profiles, absence of abortion history, burial of aborted materials, and in the case of all four private-grazing farms, complete exclusion from communal pasture, consistent with the biological expectation that targeted farm-level biosecurity can substantially reduce exposure even within a high-prevalence setting.

### 4.2 Age

Animals over 12 months of age had significantly higher seroprevalence (46.8%) than those under 12 months (23.6%), with an adjusted OR of 3.54 (95% CI: 2.15–5.97, *P* < 0.001), consistent with the progressive accumulation of environmental oocyst exposure over time. This pattern is one of the most consistently reported findings in ovine toxoplasmosis research: the nationwide Turkish survey found seropositivity of 33.92% in adults vs. 18.41% in younger animals (17), and comparable gradients have been documented in Australia (9), Italy (30), the Faroe Islands, where all seropositive animals were adults (22), Qatar (11), and inner Mongolia (21.85 vs. 10.20% in younger cohorts) (32). The increasing seropositivity with age reflects the absence of effective herd immunity rather than protective infection, a conclusion reinforced by the endemic instability demonstrated across all Turkish regions by Ceylan et al. (17), indicating that susceptible cohorts continuously enter the population without developing sterilizing immunity. While this pattern is most parsimoniously explained by cumulative horizontal exposure to environmental oocysts over time, the potential contribution of vertical (transplacental) transmission warrants consideration. Endogenous transplacental transmission has been documented in ovine *T. gondii* infection and, where prevalent, would be expected to elevate seroprevalence in young animals and thereby attenuate the age gradient (3, 4). The pronounced age effect observed here with seroprevalence in animals under 12 months less than half that of older animals suggests that horizontal transmission via environmental contamination is the dominant route in this population, rather than congenital infection. Beyond cumulative exposure time, management differences associated with age may also contribute: older breeding ewes retained across multiple seasons undergo repeated grazing cycles on potentially contaminated communal pastures and a greater number of reproductive events, whereas younger animals are more frequently housed or managed separately. There is no established evidence for an intrinsic, age-dependent physiological increase in susceptibility to *T. gondii*; the effect is therefore most plausibly attributable to prolonged and repeated environmental exposure rather than to biological susceptibility (1, 4).

### 4.3 Communal grazing

The most striking epidemiological finding was the complete absence of seropositivity among animals from the four exclusively private-grazing farms (0/40; 0%) compared with 45.5% in communal-grazing farms (232/510; Fisher's exact *P* < 0.001). Communal pastures aggregate oocyst contamination from stray and domestic cats that move freely across multiple properties, and simultaneously expose animals from different flocks to shared pasture, watering points, and feed troughs over extended periods. In Iraq, open grazing carried an OR of 9.15 (95% CI: 1.89–44.28) for seropositivity (21), and similar associations with communal or semi-extensive grazing have been documented in Algeria (33) and Tunisia (6). The unpenalized multivariable estimate for communal grazing was inflated by quasi-complete separation, as no seropositive animals occurred in the private-grazing farms (0/40 vs. 232/510, as detailed in the cross-tabulation in Table 2). After applying Firth's penalized regression, the adjusted odds ratio was 74.12 (95% CI: 10.22–9431.54). We strongly emphasize that this severe statistical instability and zero-cell sparsity represent a significant methodological restriction of our study. Although Firth's strategy successfully allowed the model to converge, it cannot overcome the fundamental data sparsity. Consequently, labeling this variable as independently linked with higher seropositivity must be approached critically; the actual magnitude of the effect (aOR = 74.12) is highly uncertain, and these wide confidence intervals should be interpreted with extreme caution as a qualitative trend rather than a precise epidemiological risk multiplier.

### 4.4 Abortion history and differential diagnosis

Farms with a recorded flock-level abortion history had a seroprevalence of 51.3 vs. 21.8% in farms without such history (crude OR = 3.79, 95% CI: 2.50–5.74; aOR = 3.77, 95% CI: 2.41–6.00; *P* < 0.001), making this the strongest independently estimable risk factor in the multivariable model. This is consistent with the established role of *T. gondii* as a primary ovine abortifacient, causing placentitis and fetal death when primary infection occurs during gestation (1, 4, 15). A critical limitation, however, is that no farm had conducted laboratory investigation of abortion events for other infectious causes, including *Neospora caninum, Brucella* spp., or *Chlamydia abortus*. This constraint is of paramount importance, as the absence of a comprehensive differential diagnosis introduces the possibility of misclassification of abortion etiologies and a potential overestimation of the reported connections between *T. gondii* exposure and reproductive failure. Therefore, these findings must be interpreted with caution. Furthermore, this diagnostic gap is not an isolated local issue but reflects a systemic deficiency in veterinary diagnostics across the country: in a separate nationwide survey of 607 Turkish veterinarians, 39.87% reported never using parasitological diagnostic methods, and 63.92% reported encountering parasitic diseases they could not diagnose, with ruminant practitioners showing significantly lower diagnostic testing rates than small animal clinicians (34). Co-infection with other abortifacient pathogens further complicates interpretation; *N. caninum* and *T. gondii* co-infections have been detected in 3.50% of Turkish sheep nationally (17), and *Coxiella burnetii* and *Chlamydia abortus* co-circulate with *T. gondii* in the broader region (35). Importantly, abortion may act not only as a consequence of infection but also as an amplifying source of within-flock transmission. Aborted fetuses, placentae, and birthing fluids from infected ewes contain high parasite loads and, when left in the environment, can contaminate pasture, feed, and water and attract definitive feline hosts, thereby perpetuating both the environmental oocyst burden and horizontal transmission to other animals (1, 6). This is consistent with the independent association observed between on-site disposal of aborted materials and seropositivity in the present study. The relevance of differential diagnosis is further underscored by the widespread distribution of *Neospora caninum* in Turkish ruminants; indeed, a recent nationwide study documented substantial *N. caninum* exposure and enzooticity specifically in small ruminant flocks across Türkiye (17, 36). The concurrent circulation of multiple abortifacient agents reinforces the need for systematic differential testing of abortion cases in Turkish flocks rather than presumptive attribution to a single pathogen. The recorded abortion history in this study therefore reflects field-level reproductive failure rather than laboratory-confirmed *T. gondii* attribution, and routine differential diagnosis for the principal ovine abortifacient agents is strongly indicated.

### 4.5 Disposal of aborted materials and barn cleaning

Leaving aborted fetuses and placentae on site was significantly associated with higher seropositivity (55.0 vs. 37.8% for burial; aOR = 2.30, 95% CI: 1.49–3.61). Infected placental material is a recognized source of environmental contamination that attracts scavenging definitive hosts, particularly stray cats, whose subsequent oocyst shedding amplifies transmission risk (1, 37). Masombuka et al. (37) reported an exceptionally high OR of 42.04 for disposal by leaving vs. burning, the highest single-variable odds ratio documented in the recent literature for this risk factor. Irregular barn cleaning was also independently associated with higher seropositivity (46.4 vs. 28.5% for weekly cleaning; aOR = 2.32, 95% CI: 1.46–3.72), likely reflecting accumulation of fecal material and contaminated bedding that increases the duration and density of oocyst exposure for housed animals; sporulated *T. gondii* oocysts are highly resistant and can remain infective in soil, water, and organic matter for many months under moist, temperate conditions (9, 38). Both variables represent directly modifiable biosecurity practices amenable to farmer education and targeted intervention.

### 4.6 Cat presence, feed and water access

Cat presence was not statistically significant in this study because cats were present on all 55 farms, with stray cats having unrestricted access to feed storage and drinking water on every farm, precluding any exposed-vs.-unexposed comparison. This reflects Turkey's broader national context: a peer-reviewed population study estimated at least 10,191 stray cats (95% CI: 8,439–11,942) in metropolitan Ankara alone (39), and the countrywide stray cat population is estimated at several million, with rural farm settings hosting free-roaming cats routinely tolerated and fed by farmers. Despite its non-significance here, the epidemiological importance of cat presence is unambiguous from the international literature. Alani and Omar (21) documented a flock-level prevalence of 81.6% in cat-exposed flocks vs. 5.9% in unexposed flocks (OR = 54.6, *P* < 0.001), and Aktaş and Aydin (15) reported that 97.73% of seropositive sheep in Erzurum had documented cat contact. A single infected cat may excrete up to 810 million oocysts over an eight-day patent period (40), and oocysts remain viable in moist environments for months to years (9). The universal cat access to feed and water documented in this study also explains the absence of significant associations with water source (well 39.4 vs. spring 46.1%; *P* = 0.116) and feed storage type (open 43.3 vs. covered 33.3%; *P* = 0.142): when oocyst contamination of feed and water is effectively universal through cat access to drinking troughs, the primary water source or storage type no longer discriminates between levels of exposure. Stray cat management, including restriction of access to feed storage, water troughs, and housing areas, therefore represents the single most impactful intervention target for reducing *T. gondii* transmission in this population and remains largely ignored.

### 4.7 Flock size

Larger flock size was associated with higher seroprevalence in univariable analysis (small < 150: 33%; medium 150–249: 41.0%; large ≥250: 49%; χ^2^ = 6.4, *P* = 0.04; OR large vs. small flock = 1.98, 95% CI: 1.14–3.46), consistent with findings from Iraq (OR = 13.1; Alani and Omar, 2025) and South Africa (37), where larger flocks were associated with greater contamination density and cumulative oocyst exposure. This association did not reach significance in the multivariable model (aOR = 1.41, *P* = 0.345), most likely because of confounding by communal grazing, which is more prevalent among larger commercial operations in Afyonkarahisar. Notably, an Italian study found the opposite direction, decreasing seropositivity with increasing flock size (41), suggesting that in highly managed systems with covered housing, per-animal dilution of cat contact may outweigh the density effect. The direction and significance of the flock size association therefore appears to depend on the broader management context, and in Afyonkarahisar the communal pasture system is the more fundamental determinant.

### 4.8 Non-significant variables

No significant association was found for sex (*P* = 0.150), consistent with most published studies (9, 11, 21). Breed was likewise non-significant (*P* = 0.117), which contrasts with earlier Turkish reports of significant breed effects (15, 16) and most likely reflects the relatively even distribution of breeds across farms with similar management in the present study. Physiological status was also non-significant (*P* = 0.243), as expected given that IgG-based ELISA reflects cumulative rather than active infection. Neither antiparasitic drug type nor treatment frequency was associated with seropositivity, consistent with the absence of any direct activity of benzimidazoles or macrocyclic lactones against *T. gondii*; rather, the routine use of anthelmintics without diagnostic confirmation reflects a broader pattern of empirical treatment documented among Turkish veterinarians (34). A potential driver of this limited diagnostic engagement was identified in a recent multinational European survey of over 1,200 ruminant stakeholders, in which Turkish respondents showed one of the highest levels of agreement (44.9%) with the statement that the costs of diagnostic testing outweigh its benefits (42). This perception may partly explain the absence of differential diagnostic investigation of abortion events observed here, and indicates that improving diagnostic uptake will require addressing both the perceived cost–benefit balance and broader practical barriers to testing

### 4.9 Public health implications

The 42.2% seroprevalence documented in this study carries direct public health relevance. *T. gondii* tissue cysts in sheep meat represent a primary human infection route. Comprehensive seroepidemiological meta-analyses consistently emphasize that high anti-*T. gondii* antibody prevalence in food-producing animals, particularly sheep, serves as a critical and robust indicator of the public health risk associated with meat consumption (1, 6, 43). Afyonkarahisar's strong traditions of fresh meat and dairy consumption create a particularly relevant exposure pathway for the local population. This is reflected in human seroepidemiological data: pregnant women from Afyonkarahisar have a documented IgG seroprevalence of 23.4% and an IgM rate of 4.3%, the latter indicating active recent transmission (13). Nationally, *T. gondii* IgG seroprevalence in Turkish pregnant women averages 36.8%, reaching 59.4% in Southeastern Anatolia (13), and the clinical consequences of congenital toxoplasmosis in Türkiye include ocular involvement (55.5% of confirmed cases), neurodevelopmental retardation (38.8%), hydrocephalus (27.7%), and a case fatality rate of 5.5% (14). These data reinforce that effective control of *T. gondii* in sheep directly contributes to human health protection, particularly for pregnant women and immunocompromised individuals, within a One Health framework.

### 4.10 Study limitations

The cross-sectional design precludes temporal inference. The complete absence of seropositivity in private-grazing farms, while epidemiologically meaningful, prevented reliable multivariable estimation of the communal grazing effect. No differential diagnostic testing for other abortifacient pathogens was performed on any farm, limiting causal attribution of abortion events to *T. gondii*. Risk factor data were collected by structured interview and are subject to recall bias. In addition, the serological approach could not differentiate between antibodies acquired through horizontal environmental exposure and those resulting from vertical transmission; the relative contribution of congenital infection to the observed age-related seroprevalence therefore remains undetermined, and paired dam–offspring or lamb-cohort studies would be required to disentangle the two transmission pathways. Because cats were present on all 55 farms, the contribution of cat presence to seropositivity could not be statistically evaluated, as the fundamental absence of variance in an exposure variable mathematically precludes the estimation of effect sizes or odds ratios in any regression model (24). More in depth studying exploring the impact on cats in the epidemiology of the disease in the region would be required. Future prospective studies incorporating differential abortifacient testing, environmental oocyst quantification, and longitudinal follow-up of seronegative private-grazing flocks would substantially strengthen the evidence base for targeted control recommendations.

## 5 Conclusions

The seroprevalence of *T. gondii* in sheep from Afyonkarahisar Province (42.2% animal-level, 80.0% farm-level) confirms high-level endemicity consistent with the national picture and with comparable semi-extensive production systems internationally. The principal independent risk factors were communal pasture use, flock-level history of abortion, leaving aborted materials on site, and irregular barn cleaning frequency; age over 12 months was also independently associated with higher seropositivity. Although cat presence could not be evaluated statistically due to its universal distribution across all farms, the unrestricted access of stray cats to feed, water, and pasture represents the primary ecological driver of *T. gondii* oocyst contamination and should be the first target of any control programme. The complete absence of laboratory-based differential diagnosis for abortifacient pathogens on any study farm, situated within the broader context of low diagnostic testing rates among ruminant veterinarians in Türkiye, highlights a systemic gap in reproductive health management. Implementing routine serological surveillance for *T. gondii, N. caninum, Brucella* spp., and *C. abortus* in abortion-reporting flocks, combined with stray cat access control, safe disposal of aborted materials, and regular barn cleaning, represents an evidence-based and operationally feasible approach to reducing both animal and human exposure to this zoonotic pathogen in Afyonkarahisar and comparable sheep-producing regions of Türkiye. Such measures are expected to have a particularly high impact on the protection of vulnerable human groups, notably pregnant women, children, and immunocompromised individuals.

## Data Availability

The original contributions presented in the study are included in the article/Supplementary material, further inquiries can be directed to the corresponding author.
