# Supplementary material for: Seroprevalence of Toxoplasma gondii infection and risk factors in sheep from Afyonkarahisar province, Türkiye
Source: Front Vet Sci. 2026 Aug 14;13:1912990. doi: 10.3389/fvets.2026.1912990 (PMC13521879; doi:10.3389/fvets.2026.1912990)
Supplement: Supplementary file 1 [file Table_1.docx]

Supplementary Material

# 1 Supplementary Data

**Supplementary File S1.**

Structured questionnaire used for flock- and animal-level data collection in the study of *Toxoplasma gondii* seroprevalence and associated risk factors in sheep, Afyonkarahisar Province, Türkiye.

**A. Farm and animal identification**

**A1.** Farm ID code ________________________

**A2.** District / Village ________________________

**A3.** GPS coordinates ________________________

**A4.** Animal ID ________________________

**A5.** Season of sampling: ☐ Spring	☐ Summer	☐ Autumn	☐ Winter

**B. Risk-factor variables (in order of presentation in Table 2)**

**1.** Age category: ☐ Under 12 months	☐ Over 12 months

**2.** Type of grazing area: ☐ Communal	☐ Private

**3.** History of abortion (flock-level): ☐ Yes	☐ No

**3a.** If yes, abortion rate (%) ________________________ *(recorded where available)*

**4.** Disposal of aborted materials: ☐ Buried	☐ Left on site

**5.** Cleaning frequency: ☐ Weekly	☐ Irregular

**6.** Presence of dogs on farm: ☐ Yes	☐ No

**7.** Main roughage type: ☐ Hay	☐ Straw

**8.** Sex: ☐ Female	☐ Male

**9.** Breed: ☐ Pırlak	☐ Merino	☐ Akkaraman	☐ Crossbreed

**10.** Physiological status: ☐ Pregnant	☐ Lactating	☐ Dry

**11.** Grazing system (daily grazing duration): ☐ Full-day (~12 h) ☐ Extended (>12 h)

**12.** Water source: ☐ Well / Borehole	☐ Surface / Spring

**13.** Feed storage: ☐ Open	☐ Covered

**14.** Cat presence type: ☐ Stray only	☐ Both (owned + stray)

**15.** Cats in housing: ☐ Yes	☐ No

**16.** Rodent control: ☐ Yes	☐ No

**17.** Antiparasitic drug type: ☐ Benzimidazoles	☐ Macrocyclic lactones

**18.** Production purpose: ☐ Meat	☐ Mixed

**19.** Total flock size (number of sheep): ________________________ *(categorised: Small <150 / Medium 150–249 / Large ≥250)*

**C. Abortifacient differential testing**

**C1.** Laboratory investigation of abortion for other abortifacient agents (Neospora / Brucella / Chlamydia) performed? ☐ Yes	☐ No
